# Supplementary material for: New Frontiers in Autoimmune Diagnostics: A Systematic Review on Saliva Testing
Source: Int J Environ Res Public Health. 2023 May 10;20(10):5782. doi: 10.3390/ijerph20105782 (PMC10218106; doi:10.3390/ijerph20105782)
Supplement: Supplementary file 1 [file ijerph-20-05782-s001.zip › ijerph-2310673-supplementary.pdf]

Table S1. Sensitivity and specificity from all the selected studies. UK: unknown.

	Authors, Year of Publication	Sensibility	Specificity
CTDs	Zhang [23]	UK	UK
CTDs	Sciascia [24]	UK	UK
Sicca syndrome	Ching [25]	Ro60: 70%, Ro52: 67%	Ro60: 96%; Ro52: 100%
Sicca syndrome	Burbelo [26]	UK	UK
RA	Demoruelle [27]	UK	UK
RA	Ljungberg [28]	UK	UK
RA	Svärd 2020 [29]	UK	UK
RA	Svärd 2019 [30]	UK	UK
PV	Koopaie [31]	UK	UK
PB	Esmaili [32]	BP180: Serum/saliva 88% vs. 87%; BP230 serum/saliva 48% vs. 77%	BP180 saliva/serum: 96% vs. 96%; BP230: serum/saliva 96% vs. 62%.
PV	Hallaji [33]	Dsg1 sensitivity serum/saliva: 72/70%; Dsg3 sensitivity serum/saliva: 94/94%	UK
DM	Todd [34]	UK	UK
DM	Markopulos [35]	UK	UK
DM	Tiberti [36]	UK	UK
PBC	Lu [37]	81.82%	80%
PBC	Palmer [38]	UK	UK
PBC	Ikuno [39]	UK	UK
Celiac disease	Ajdani [40]	98.15%	80%
